# Poly[bis[μ-1,3-bis­(imidazol-1-ylmeth­yl)benzene-κ^2^ *N* ^3^:*N* ^3′^]bis­(nitrato-κ*O*)cadmium]

**DOI:** 10.1107/S1600536811021027

**Published:** 2011-06-11

**Authors:** Xi-Ying Hu, Guang-Rui Yang, Wen-Wen Shan

**Affiliations:** a Institute of Environmental and Municipal Engineering, North China University of Water Conservancy and Electric Power, Zhengzhou 450011, People’s Republic of China

## Abstract

A novel metal–organic framework based on 1,3-bis­(imidazol-1-ylmeth­yl)benzene (1,3-bimb), [Cd(NO_3_)_2_(C_14_H_14_N_4_)_2_]_*n*_, has been synthesized hydro­thermally. The structure exhibits a two-dimensional metal–organic (4,4)-net composed of Cd^II^ atoms and bimb ligands, and such layers are further joined through inter­layer C—H⋯O hydrogen bonds to generate a three-dimensional supra­molecular structure.

## Related literature

For background to the network topologies and applications of coordination polymers, see: Maspoch *et al.* (2007 ▶); Ockwig *et al.* (2005 ▶); Zang *et al.* (2006 ▶); Zhang *et al.* (2009 ▶). For synthesis and related structures with the bimb ligand, see: Hoskins *et al.* (1997 ▶). For C—H⋯O hydrogen bonds, see: Desiraju (1996 ▶); Broder *et al.* (2002 ▶). 
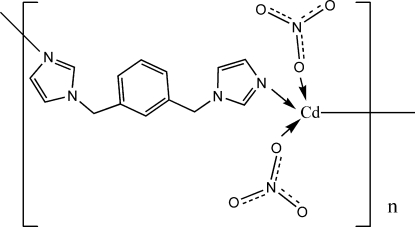


## Experimental

### 

#### Crystal data

[Cd(NO_3_)_2_(C_14_H_14_N_4_)_2_]
*M* *_r_* = 713.00
Monoclinic, 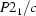

*a* = 8.4542 (8) Å
*b* = 19.3910 (18) Å
*c* = 9.2222 (8) Å
β = 102.415 (10)°
*V* = 1476.5 (2) Å^3^
*Z* = 2
Mo *K*α radiation
μ = 0.80 mm^−1^
*T* = 296 K
0.21 × 0.20 × 0.19 mm

#### Data collection

Bruker SMART APEXII CCD area-detector diffractometer
Absorption correction: multi-scan (*SADABS*; Bruker, 2005 ▶) *T*_min_ = 0.850, *T*_max_ = 0.863
5606 measured reflections
2578 independent reflections
2253 reflections with *I* > 2σ(*I*)
*R*_int_ = 0.017

#### Refinement

*R*[*F* ^2^ > 2σ(*F* ^2^)] = 0.044
*wR*(*F* ^2^) = 0.123
*S* = 1.06
2578 reflections
199 parameters
1 restraint
H-atom parameters constrained
Δρ_max_ = 1.95 e Å^−3^
Δρ_min_ = −0.86 e Å^−3^

### 

Data collection: *APEX2* (Bruker, 2005 ▶); cell refinement: *SAINT* (Bruker, 2005 ▶); data reduction: *SAINT*; program(s) used to solve structure: *SHELXS97* (Sheldrick, 2008 ▶); program(s) used to refine structure: *SHELXL97* (Sheldrick, 2008 ▶); molecular graphics: *DIAMOND* (Brandenburg, 2010 ▶); software used to prepare material for publication: *SHELXTL* (Sheldrick, 2008 ▶).

## Supplementary Material

Crystal structure: contains datablock(s) I, global. DOI: 10.1107/S1600536811021027/hp2007sup1.cif

Structure factors: contains datablock(s) I. DOI: 10.1107/S1600536811021027/hp2007Isup2.hkl

Additional supplementary materials:  crystallographic information; 3D view; checkCIF report

## Figures and Tables

**Table 1:** Hydrogen-bond geometry (Å, °)

*D*—H⋯*A*	*D*—H	H⋯*A*	*D*⋯*A*	*D*—H⋯*A*
C3—H3⋯O2^i^	0.93	2.71	3.525 (8)	147
C4—H4*B*⋯O1^i^	0.97	2.67	3.525 (6)	148
C4—H4*B*⋯O2^i^	0.97	2.83	3.633 (9)	141
C10—H10⋯O2^i^	0.93	2.63	3.506 (9)	158
C11—H11*A*⋯O1^ii^	0.97	2.70	3.518 (7)	142
C12—H12⋯O1^ii^	0.93	2.37	3.210 (6)	151

Symmetry codes: (i) 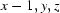; (ii) 
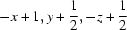
.

**Fig. 1.**
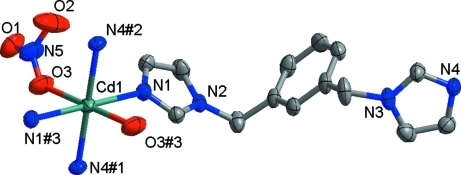
Metal coordination and atom labeling in title compound (thermal ellipsoids at 50% probability level). All H atoms are omitted for clarity.

**Fig. 2.**
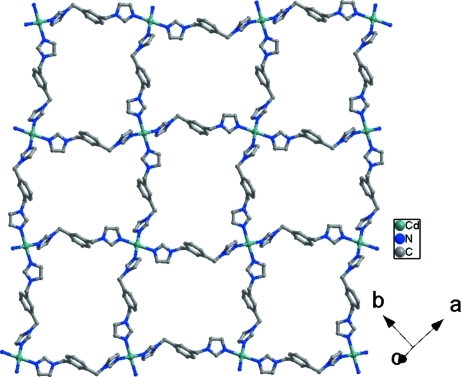
A view of the layer structure in compound 1. Cd atoms are drawn as polyhedra.

**Fig. 3.**
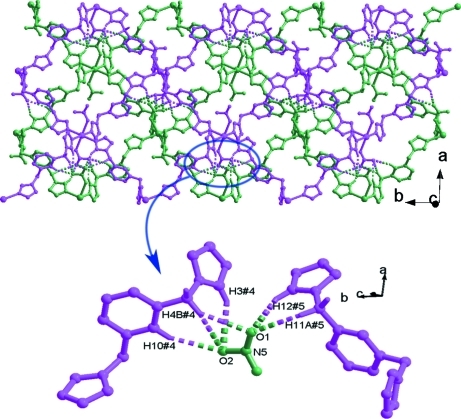
The three-dimensional supramolecular structure connected by interlayer C—H···O hydrogen bonds. Dotted lines represent C—H···O bonds. Symmetry codes: #4 x + 1, y, z; #5 -x + 1, y - 1/2, -z + 1/2.

## supplementary crystallographic information

### Comment

Extensive research has been focused on the supramolecular coordination assemblies not only for their variety of architectures but also for the potential applications as functional materials (Maspoch *et al.*, 2007; Ockwig *et al.*, 2005). Many imidazole-containing ligands have been successfully employed in the generation of many interesting systems in possession of multidimensional networks and properties (Zang *et al.*, 2006; Zhang *et al.*, 2009). To further explore various factors that influence the formation of result structures in the assembly reactions, we undertake synthetic and structural studies on one novel Cd^II^ coordination polymers based on the highly flexible bidentate ligand 1,3-bis(imidazol-1-ylmethyl)-benzene (1,3-bimb): [Cd(bimb)_2_(NO_3_)_2_]*_n_* (1).

X-ray crystallographic analysis revealed that 1 crystallizes in monoclinic space group *P*2_1_/*c*. As depicted in Fig. 1, the asymmetric unit consists of a half Cd^II^ atom, one bimb ligand and one nitrate ion. Each Cd ion is in a slightly elongated octahedral coordination environment and coordinated by four N atoms from different bimb ligands and two O atoms from two nitrate ions. Four N atoms comprise the equatorial plane, while two O atoms occupy the axial positions. Each bimb ligand acts as a µ_2_-bridge in *trans*-conformation with the dihedral angle of the two imidazole rings being *ca* 68.40 (16)°. Adjacent metal atoms are bridged by bimb ligands from two directions which are almost mutually perpendicular to form a (4,4)-net with the Cd···Cd distances of *ca* 14.2654 (10) Å (Fig. 2). The coordinated nitrate ions hang from the layer. Neighboring layers are arranged parallel with the uncoordinated O atoms closed to some H atoms from adjacent layer, and a number of interlayer C—H···O hydrogen bonds can be detected which contribute to the formation of the three-dimensional supromolecular structure, as shown in Fig. 3. The hydrogen-bonding geometry is listed in Table 1.

### Experimental

1,3-bis(imidazol-1-ylmethyl)-benzene (bimb) was prepared according to the literature (Hoskins *et al.*, 1997), all other starting materials were of analytical grade and obtained from commercial sources without further purification. Compound 1 was synthesized hydrothermally in a Teflon-lined stainless steel container by heating a mixture of 1,3-bis(imidazol-1-ylmethyl)-benzene (bimb) (0.0119 g, 0.05 mmol), Cd(NO_3_)_2_.4H_2_O (0.0154 g, 0.05 mmol) and KOH (0.0056 g, 0.1 mmol) in 7 ml of distilled water at 120°C for 3 d, and then cooled to room temperature. Colorless rectangular crystals of 1 were obtained in 79% yield based on Cd.

### Refinement

H atoms were positioned geometrically and refined using a riding model, with C—H = 0.93 Å, *U*_iso_(H) = 1.2U_eq_(C) for aromatic H, and C—H = 0.97 Å, *U*_iso_(H) = 1.2U_eq_(C) for CH_2_.

### Crystal data

**Table:**

[Cd(NO_3_)_2_(C_14_H_14_N_4_)_2_]	*F*(000) = 724
*M**_r_* = 713.00	*D*_x_ = 1.604 Mg m^−^^3^
Monoclinic, *P*2_1_/*c*	Mo *K*α radiation, λ = 0.71073 Å
Hall symbol: -P 2ybc	Cell parameters from 3957 reflections
*a* = 8.4542 (8) Å	θ = 3.1–29.1°
*b* = 19.3910 (18) Å	µ = 0.80 mm^−^^1^
*c* = 9.2222 (8) Å	*T* = 296 K
β = 102.415 (10)°	Needle, colourless
*V* = 1476.5 (2) Å^3^	0.21 × 0.20 × 0.19 mm
*Z* = 2	

### Data collection

**Table:**

Bruker SMART APEXII CCD area-detector diffractometer	2578 independent reflections
Radiation source: fine-focus sealed tube	2253 reflections with *I* > 2σ(*I*)
graphite	*R*_int_ = 0.017
ω scans	θ_max_ = 25.0°, θ_min_ = 3.1°
Absorption correction: multi-scan (*SADABS*; Bruker, 2005)	*h* = −10→6
*T*_min_ = 0.850, *T*_max_ = 0.863	*k* = −21→23
5606 measured reflections	*l* = −10→10

### Refinement

**Table:**

Refinement on *F*^2^	Primary atom site location: structure-invariant direct methods
Least-squares matrix: full	Secondary atom site location: difference Fourier map
*R*[*F*^2^ > 2σ(*F*^2^)] = 0.044	Hydrogen site location: inferred from neighbouring sites
*wR*(*F*^2^) = 0.123	H-atom parameters constrained
*S* = 1.06	*w* = 1/[σ^2^(*F*_o_^2^) + (0.0689*P*)^2^ + 2.589*P*] where *P* = (*F*_o_^2^ + 2*F*_c_^2^)/3
2578 reflections	(Δ/σ)_max_ < 0.001
199 parameters	Δρ_max_ = 1.95 e Å^−^^3^
1 restraint	Δρ_min_ = −0.86 e Å^−^^3^

### Special details

**Table:**

Geometry. All e.s.d.'s (except the e.s.d. in the dihedral angle between two l.s. planes) are estimated using the full covariance matrix. The cell e.s.d.'s are taken into account individually in the estimation of e.s.d.'s in distances, angles and torsion angles; correlations between e.s.d.'s in cell parameters are only used when they are defined by crystal symmetry. An approximate (isotropic) treatment of cell e.s.d.'s is used for estimating e.s.d.'s involving l.s. planes.
Refinement. Refinement of *F*^2^ against ALL reflections. The weighted *R*-factor *wR* and goodness of fit *S* are based on *F*^2^, conventional *R*-factors *R* are based on *F*, with *F* set to zero for negative *F*^2^. The threshold expression of *F*^2^ > σ(*F*^2^) is used only for calculating *R*-factors(gt) *etc*. and is not relevant to the choice of reflections for refinement. *R*-factors based on *F*^2^ are statistically about twice as large as those based on *F*, and *R*-factors based on ALL data will be even larger.

### Fractional atomic coordinates and isotropic or equivalent isotropic displacement parameters (Å^2^)

**Table:**

	*x*	*y*	*z*	*U*_iso_*/*U*_eq_	
Cd1	0.5000	0.5000	0.0000	0.03330 (18)	
O1	0.9948 (4)	0.4485 (2)	0.2556 (4)	0.0657 (10)	
O2	0.8878 (9)	0.5415 (3)	0.1617 (11)	0.183 (4)	
O3	0.7504 (4)	0.45152 (19)	0.1259 (4)	0.0626 (7)	
N1	0.4529 (4)	0.55084 (17)	0.2160 (4)	0.0364 (7)	
N2	0.3328 (4)	0.57252 (16)	0.4000 (3)	0.0322 (7)	
N3	−0.2702 (4)	0.81383 (18)	0.3469 (4)	0.0401 (8)	
N4	−0.4073 (4)	0.89430 (17)	0.4339 (4)	0.0379 (8)	
N5	0.8818 (5)	0.4775 (3)	0.1699 (6)	0.0626 (7)	
C1	0.5664 (5)	0.5731 (2)	0.3353 (5)	0.0419 (10)	
H1	0.6763	0.5781	0.3374	0.050*	
C2	0.4946 (5)	0.5868 (2)	0.4507 (5)	0.0463 (11)	
H2	0.5446	0.6026	0.5446	0.056*	
C3	0.3138 (5)	0.5521 (2)	0.2602 (4)	0.0342 (8)	
H3	0.2145	0.5400	0.2002	0.041*	
C4	0.2053 (6)	0.5788 (2)	0.4856 (5)	0.0414 (10)	
H4A	0.2443	0.5598	0.5841	0.050*	
H4B	0.1116	0.5521	0.4373	0.050*	
C5	0.1556 (5)	0.6528 (2)	0.4988 (4)	0.0335 (8)	
C6	0.2395 (5)	0.6941 (2)	0.6119 (5)	0.0436 (10)	
H6	0.3224	0.6753	0.6839	0.052*	
C7	0.2009 (5)	0.7630 (2)	0.6186 (5)	0.0469 (11)	
H7	0.2587	0.7904	0.6947	0.056*	
C8	0.0777 (6)	0.7914 (2)	0.5137 (5)	0.0440 (10)	
H8	0.0538	0.8381	0.5178	0.053*	
C9	−0.0113 (5)	0.7503 (2)	0.4012 (4)	0.0379 (9)	
C10	0.0285 (5)	0.6813 (2)	0.3951 (4)	0.0348 (8)	
H10	−0.0307	0.6535	0.3206	0.042*	
C11	−0.1437 (6)	0.7816 (3)	0.2829 (5)	0.0509 (12)	
H11A	−0.0969	0.8160	0.2283	0.061*	
H11B	−0.1919	0.7459	0.2136	0.061*	
C12	−0.2881 (5)	0.8817 (2)	0.3668 (5)	0.0410 (10)	
H12	−0.2241	0.9155	0.3368	0.049*	
C13	−0.4682 (5)	0.8310 (2)	0.4589 (5)	0.0430 (10)	
H13	−0.5539	0.8236	0.5056	0.052*	
C14	−0.3855 (5)	0.7810 (2)	0.4059 (5)	0.0469 (11)	
H14	−0.4031	0.7337	0.4088	0.056*	

### Atomic displacement parameters (Å^2^)

**Table:**

	*U*^11^	*U*^22^	*U*^33^	*U*^12^	*U*^13^	*U*^23^
Cd1	0.0367 (3)	0.0294 (3)	0.0390 (3)	−0.00351 (15)	0.01983 (19)	−0.00143 (16)
O1	0.0330 (16)	0.089 (3)	0.072 (2)	0.0071 (17)	0.0045 (16)	0.018 (2)
O2	0.124 (5)	0.077 (2)	0.302 (10)	−0.021 (4)	−0.058 (6)	0.044 (5)
O3	0.0348 (12)	0.0655 (16)	0.082 (2)	−0.0069 (12)	0.0001 (13)	0.0074 (15)
N1	0.0393 (18)	0.0353 (17)	0.0387 (18)	0.0008 (15)	0.0175 (15)	−0.0031 (15)
N2	0.0369 (17)	0.0287 (16)	0.0330 (17)	0.0082 (14)	0.0121 (14)	0.0015 (13)
N3	0.0437 (19)	0.0371 (18)	0.0404 (19)	0.0151 (16)	0.0112 (16)	−0.0011 (15)
N4	0.0347 (17)	0.0343 (18)	0.049 (2)	0.0069 (14)	0.0172 (15)	−0.0008 (15)
N5	0.0348 (12)	0.0655 (16)	0.082 (2)	−0.0069 (12)	0.0001 (13)	0.0074 (15)
C1	0.031 (2)	0.040 (2)	0.056 (3)	0.0006 (18)	0.0114 (19)	−0.007 (2)
C2	0.045 (3)	0.051 (3)	0.039 (2)	0.005 (2)	0.0020 (19)	−0.013 (2)
C3	0.035 (2)	0.032 (2)	0.037 (2)	0.0019 (16)	0.0102 (16)	0.0000 (16)
C4	0.054 (3)	0.037 (2)	0.040 (2)	0.0109 (19)	0.025 (2)	0.0086 (18)
C5	0.037 (2)	0.037 (2)	0.032 (2)	0.0080 (17)	0.0188 (16)	0.0031 (16)
C6	0.039 (2)	0.060 (3)	0.033 (2)	0.011 (2)	0.0090 (18)	−0.0037 (19)
C7	0.043 (2)	0.050 (3)	0.049 (3)	−0.003 (2)	0.013 (2)	−0.018 (2)
C8	0.049 (3)	0.033 (2)	0.056 (3)	0.0035 (19)	0.025 (2)	−0.0064 (19)
C9	0.042 (2)	0.041 (2)	0.035 (2)	0.0125 (18)	0.0183 (18)	−0.0013 (18)
C10	0.038 (2)	0.035 (2)	0.035 (2)	0.0029 (17)	0.0160 (17)	−0.0055 (17)
C11	0.062 (3)	0.055 (3)	0.039 (2)	0.031 (2)	0.018 (2)	−0.001 (2)
C12	0.042 (2)	0.033 (2)	0.054 (3)	0.0082 (18)	0.022 (2)	0.0057 (19)
C13	0.036 (2)	0.041 (2)	0.054 (3)	0.0010 (18)	0.0125 (19)	0.004 (2)
C14	0.047 (2)	0.033 (2)	0.057 (3)	0.0044 (19)	0.004 (2)	0.001 (2)

### Geometric parameters (Å, °)

**Table:**

Cd1—N4^i^	2.322 (3)	C2—H2	0.9300
Cd1—N4^ii^	2.322 (3)	C3—H3	0.9300
Cd1—N1^iii^	2.332 (3)	C4—C5	1.508 (5)
Cd1—N1	2.332 (3)	C4—H4A	0.9700
Cd1—O3	2.378 (3)	C4—H4B	0.9700
Cd1—O3^iii^	2.378 (3)	C5—C6	1.384 (6)
O1—N5	1.236 (6)	C5—C10	1.389 (6)
O2—N5	1.247 (8)	C6—C7	1.379 (6)
O3—N5	1.207 (5)	C6—H6	0.9300
N1—C3	1.326 (5)	C7—C8	1.376 (7)
N1—C1	1.365 (5)	C7—H7	0.9300
N2—C3	1.326 (5)	C8—C9	1.394 (6)
N2—C2	1.375 (5)	C8—H8	0.9300
N2—C4	1.472 (5)	C9—C10	1.384 (6)
N3—C12	1.341 (5)	C9—C11	1.512 (6)
N3—C14	1.371 (6)	C10—H10	0.9300
N3—C11	1.468 (5)	C11—H11A	0.9700
N4—C12	1.314 (5)	C11—H11B	0.9700
N4—C13	1.369 (6)	C12—H12	0.9300
N4—Cd1^iv^	2.322 (3)	C13—C14	1.347 (6)
C1—C2	1.360 (6)	C13—H13	0.9300
C1—H1	0.9300	C14—H14	0.9300
			
N4^i^—Cd1—N4^ii^	180.0	N2—C4—C5	111.8 (3)
N4^i^—Cd1—N1^iii^	91.13 (12)	N2—C4—H4A	109.3
N4^ii^—Cd1—N1^iii^	88.87 (12)	C5—C4—H4A	109.3
N4^i^—Cd1—N1	88.87 (12)	N2—C4—H4B	109.3
N4^ii^—Cd1—N1	91.13 (12)	C5—C4—H4B	109.3
N1^iii^—Cd1—N1	180.00 (15)	H4A—C4—H4B	107.9
N4^i^—Cd1—O3	99.31 (12)	C6—C5—C10	118.9 (4)
N4^ii^—Cd1—O3	80.69 (12)	C6—C5—C4	120.4 (4)
N1^iii^—Cd1—O3	87.27 (13)	C10—C5—C4	120.7 (4)
N1—Cd1—O3	92.73 (13)	C7—C6—C5	120.5 (4)
N4^i^—Cd1—O3^iii^	80.69 (12)	C7—C6—H6	119.8
N4^ii^—Cd1—O3^iii^	99.31 (12)	C5—C6—H6	119.8
N1^iii^—Cd1—O3^iii^	92.73 (13)	C8—C7—C6	120.5 (4)
N1—Cd1—O3^iii^	87.27 (13)	C8—C7—H7	119.8
O3—Cd1—O3^iii^	180.0	C6—C7—H7	119.8
N5—O3—Cd1	131.1 (3)	C7—C8—C9	120.0 (4)
C3—N1—C1	105.2 (3)	C7—C8—H8	120.0
C3—N1—Cd1	126.6 (3)	C9—C8—H8	120.0
C1—N1—Cd1	127.1 (3)	C10—C9—C8	119.1 (4)
C3—N2—C2	107.2 (3)	C10—C9—C11	120.5 (4)
C3—N2—C4	126.6 (4)	C8—C9—C11	120.3 (4)
C2—N2—C4	126.2 (4)	C9—C10—C5	121.0 (4)
C12—N3—C14	106.9 (3)	C9—C10—H10	119.5
C12—N3—C11	125.9 (4)	C5—C10—H10	119.5
C14—N3—C11	127.1 (4)	N3—C11—C9	111.8 (3)
C12—N4—C13	105.4 (3)	N3—C11—H11A	109.3
C12—N4—Cd1^iv^	128.7 (3)	C9—C11—H11A	109.3
C13—N4—Cd1^iv^	125.9 (3)	N3—C11—H11B	109.3
O3—N5—O1	123.7 (5)	C9—C11—H11B	109.3
O3—N5—O2	116.2 (5)	H11A—C11—H11B	107.9
O1—N5—O2	117.1 (5)	N4—C12—N3	111.6 (4)
C2—C1—N1	109.8 (4)	N4—C12—H12	124.2
C2—C1—H1	125.1	N3—C12—H12	124.2
N1—C1—H1	125.1	C14—C13—N4	109.9 (4)
C1—C2—N2	105.9 (4)	C14—C13—H13	125.0
C1—C2—H2	127.1	N4—C13—H13	125.0
N2—C2—H2	127.1	C13—C14—N3	106.2 (4)
N1—C3—N2	112.0 (4)	C13—C14—H14	126.9
N1—C3—H3	124.0	N3—C14—H14	126.9
N2—C3—H3	124.0		
			
N4^i^—Cd1—O3—N5	13.2 (5)	N2—C4—C5—C6	−86.3 (5)
N4^ii^—Cd1—O3—N5	−166.8 (5)	N2—C4—C5—C10	91.6 (5)
N1^iii^—Cd1—O3—N5	103.9 (5)	C10—C5—C6—C7	−2.1 (6)
N1—Cd1—O3—N5	−76.1 (5)	C4—C5—C6—C7	175.8 (4)
N4^i^—Cd1—N1—C3	124.0 (3)	C5—C6—C7—C8	0.5 (7)
N4^ii^—Cd1—N1—C3	−56.0 (3)	C6—C7—C8—C9	1.3 (7)
O3—Cd1—N1—C3	−136.7 (3)	C7—C8—C9—C10	−1.4 (6)
O3^iii^—Cd1—N1—C3	43.3 (3)	C7—C8—C9—C11	−178.2 (4)
N4^i^—Cd1—N1—C1	−69.9 (3)	C8—C9—C10—C5	−0.2 (6)
N4^ii^—Cd1—N1—C1	110.1 (3)	C11—C9—C10—C5	176.6 (4)
O3—Cd1—N1—C1	29.4 (3)	C6—C5—C10—C9	2.0 (6)
O3^iii^—Cd1—N1—C1	−150.6 (3)	C4—C5—C10—C9	−175.9 (4)
Cd1—O3—N5—O1	167.3 (4)	C12—N3—C11—C9	102.7 (5)
Cd1—O3—N5—O2	7.5 (9)	C14—N3—C11—C9	−72.9 (6)
C3—N1—C1—C2	0.6 (5)	C10—C9—C11—N3	123.7 (4)
Cd1—N1—C1—C2	−167.8 (3)	C8—C9—C11—N3	−59.5 (6)
N1—C1—C2—N2	0.0 (5)	C13—N4—C12—N3	0.3 (5)
C3—N2—C2—C1	−0.6 (5)	Cd1^iv^—N4—C12—N3	−179.9 (3)
C4—N2—C2—C1	179.4 (4)	C14—N3—C12—N4	−0.2 (5)
C1—N1—C3—N2	−1.0 (4)	C11—N3—C12—N4	−176.6 (4)
Cd1—N1—C3—N2	167.5 (2)	C12—N4—C13—C14	−0.3 (5)
C2—N2—C3—N1	1.0 (5)	Cd1^iv^—N4—C13—C14	179.9 (3)
C4—N2—C3—N1	−178.9 (3)	N4—C13—C14—N3	0.2 (5)
C3—N2—C4—C5	−102.7 (5)	C12—N3—C14—C13	0.0 (5)
C2—N2—C4—C5	77.3 (5)	C11—N3—C14—C13	176.3 (4)

Symmetry codes: (i) *x*+1, −*y*+3/2, *z*−1/2; (ii) −*x*, *y*−1/2, −*z*+1/2; (iii) −*x*+1, −*y*+1, −*z*; (iv) −*x*, *y*+1/2, −*z*+1/2.

### Hydrogen-bond geometry (Å, °)

**Table:**

*D*—H···*A*	*D*—H	H···*A*	*D*···*A*	*D*—H···*A*
C3—H3···O2^v^	0.93	2.71	3.525 (8)	147.
C4—H4B···O1^v^	0.97	2.67	3.525 (6)	148.
C4—H4B···O2^v^	0.97	2.83	3.633 (9)	141.
C10—H10···O2^v^	0.93	2.63	3.506 (9)	158.
C11—H11A···O1^vi^	0.97	2.70	3.518 (7)	142.
C12—H12···O1^vi^	0.93	2.37	3.210 (6)	151.

Symmetry codes: (v) *x*−1, *y*, *z*; (vi) −*x*+1, *y*+1/2, −*z*+1/2.
